# Fear of Falling: 30 Years of Research

**DOI:** 10.1093/geroni/igaf122.3462

**Published:** 2025-12-31

**Authors:** Helen Lach, Joseph Trinitas

**Affiliations:** Saint Louis University, St. Louis, Missouri, United States; Saint Louis University, St. Louis, Missouri, United States

## Abstract

Fear of falling has the potential to reduce activity and quality of life for community-dwelling older people, and an increased risk of falling. This topic has been explored for over 30 years. As a result, the aim of this umbrella review is to explore the state of the science on fear of falling to determine implications for future research. We searched databases including CINAHL, Medline (OVID), Scopus, and Cochrane for reviews (systematic, integrative, metanalyses, metasyntheses) since 1990. Studies with a focus on fear of falling in community-dwelling older adults as the primary topic or outcome in English were included, resulting in 879 articles. After removing duplicates, 606 articles remained for title and abstract screening. Studies of specific conditions such as stroke or specific settings (e.g. nursing home) were excluded. Data was extracted to examine designs, samples, measures, and outcomes of reviews of descriptive and experimental studies for the final 47 articles. Overall, 30 years of evidence confirms that that fear of falling is a significant public health problem for older people, common even among those who have not had a fall. Fear of falling leads to negative outcomes including reduced activity, depression and increased risk of nursing home placement, and is a risk factor for future falls. Further evidence supports exercise and multifactorial interventions to reduce fear of falling and improve quality of life for older people. Future research could help target interventions to improve precision of outcomes.

